# Supplementary material for: Meta-Analysis and Experimental Evidence Reveal No Impact of Nosema ceranae Infection on Honeybee Carbohydrate Consumption
Source: Microb Ecol. 2025 May 30;88(1):56. doi: 10.1007/s00248-025-02550-z (PMC12122660; doi:10.1007/s00248-025-02550-z)
Supplement: Supplementary file 1 — Supplementary file1 (DOCX 1260 KB) [file 248_2025_2550_MOESM1_ESM.docx]

Supplementary Material 1

**Meta-analysis and experimental evidence reveal no impact of Nosema ceranae infection on honeybee carbohydrate consumption**

Monika Ostap-Chec^1*^, Weronika Antoł^1^, Daniel Bajorek^1^, Ewelina Berbeć^2^, Dawid Moroń^1^, Marcin Rapacz^3^, Krzysztof Miler^1*^

^1^ Institute of Systematics and Evolution of Animals of the Polish Academy of Sciences, Kraków, Poland

^2^ Department of Bees Breeding, Institute of Animal Husbandry and Breeding, Wroclaw University of Environmental and Life Sciences, Wrocław, Poland

^3^ Department of Plant Breeding, Physiology, and Seed Science, University of Agriculture in Kraków, Poland

*****Correspondence:

E-mails:
ostap.chec@isez.pan.krakow.pl (Monika Ostap-Chec),
miler@isez.pan.krakow.pl (Krzysztof Miler)


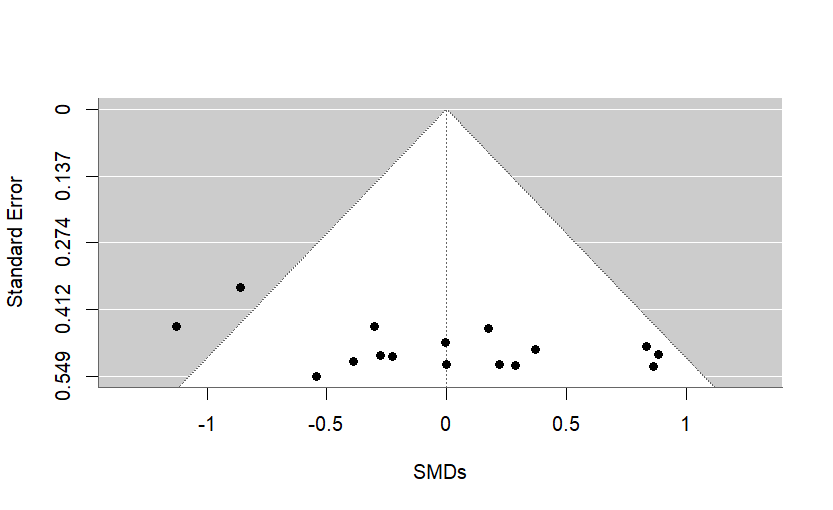


**Supplementary Figure 1**. Funnel plot (standard error plotted against SMDs) for the studies included in the meta-analysis. Each dot represents one study.
